# A New Semi-Quantum Two-Way Authentication Protocol between Control Centers and Neighborhood Gateways in Smart Grids

**DOI:** 10.3390/e26080644

**Published:** 2024-07-29

**Authors:** Qiandong Zhang, Kejia Zhang, Kunchi Hou, Long Zhang

**Affiliations:** 1 School of Mathematical Science, Heilongjiang University, Harbin 150080, China; qiandongzhang@163.com (Q.Z.); houkunchi@hlju.edu.cn (K.H.); lzhang@hlju.edu.cn (L.Z.); 2 State Key Laboratory of Public Big Data, Guizhou University, Guiyang 550000, China

**Keywords:** quantum cryptography, semi-quantum, identity authentication, single photon, smart grid

## Abstract

To address the potential threat to the power grid industry posed by quantum computers and ensure the security of bidirectional communication in smart grids, it is imperative to develop quantum-safe authentication protocols. This paper proposes a semi-quantum bidirectional authentication protocol between a control center (CC) and a neighboring gateway (NG). This method uses single photons to facilitate communication between the CC and the NG. Security analysis demonstrates that the protocol can effectively resist common attack methods, including double CNOT attacks, impersonation attacks, interception-measurement-retransmission attacks, and entanglement-measurement attacks. Comparisons with other protocols reveal that this protocol has significant advantages, making it more appealing and practical for real-world applications. Finally, by simulating the protocol on the IBM quantum simulator, this protocol not only validates the theoretical framework but also confirms the practical feasibility of the protocol.

## 1. Introduction

Smart grids revolutionize lifestyles by employing cutting-edge technologies, optimizing grid performance, dependability, and promoting the widespread adoption of renewable energy sources [1,2]. However, their complex interconnections and incorporation of advanced software and hardware systems make smart grids vulnerable to a variety of security threats. The challenges include identity forgery, unauthorized access, data privacy infringements, and denial-of-service assaults. These challenges have the potential to cause security concerns such as power system failures, user data breaches, and financial losses [3,4,5].

**Figure 1:**
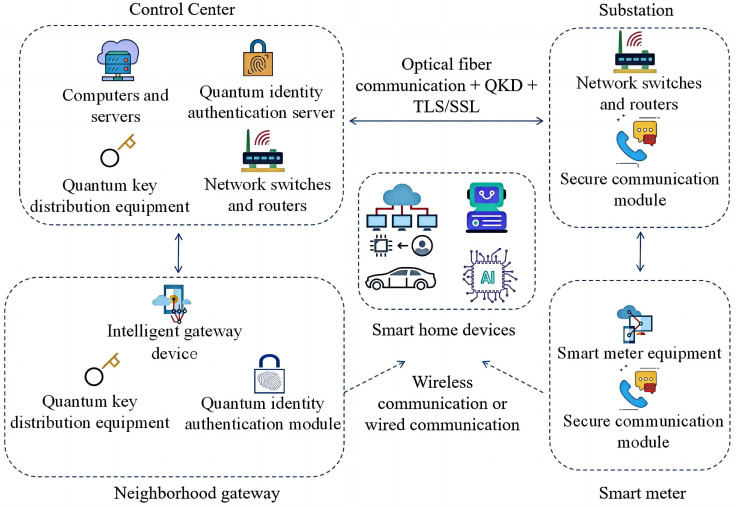
Smart grid architecture.

Currently, the aforementioned security challenges are primarily addressed using traditional encryption and authentication methods [6,7]. However, most of these methods depend on fundamental mathematical problems such as large integer factorization and discrete logarithm problems, which are susceptible to quantum computer attacks [8,9]. To mitigate this issue, researchers have proposed incorporating quantum technology into smart power systems to enhance the security of smart power networks. Quantum technology offers unique advantages in smart grids. First, quantum key distribution (QKD) technology is used to distribute keys, effectively preventing information from being eavesdropped on or tampered with [10]. Second, quantum authentication technology is used for efficient identity verification, ensuring the security and integrity of communications and data exchange [11]. Research indicates that the inability to establish effective identity authentication between senders and receivers is a primary factor causing security risks in smart grids [12,13,14]. Therefore, integrating quantum-based identity authentication into smart grids is crucial. This integration can provide highly secure communications while meeting the complex operational and management needs of smart grids, such as remote control, smart energy trading, and user privacy protection, as shown in Figure 1.

Since Crepeau proposed the first quantum identity authentication protocol [15] in 1997, development of protocols based on both entangled and non-entangled states have continued [16,17,18,19]. In practice, implementing devices with quantum capabilities, especially in smart grids, is both expensive and impractical. Thus, in practical application of quantum cryptography it is very important to limit the quantum capabilities of participants. In 2007, Boyer introduced the half-quantum concept to improve the practical implementation of quantum cryptographic protocols [20]. The half-quantum concept allows some participants to have partial quantum capabilities: (1) measuring particles using the Z basis; (2) preparing Z-based particles; (3) rearranging the positions of particles; and (4) directly returning particles. Subsequently, a number of half-quantum cryptographic protocols have been proposed for various tasks [21,22,23,24,25,26,27,28].

As an important part of the modern power system, the security of the smart grid directly affects the reliable supply of electricity and stable operation of the system. Because smart grids involve many devices and nodes, traditional quantum cryptographic protocols may encounter high costs and complexity in practical applications. Semi-quantum cryptographic protocols provide a solution that reduces equipment complexity and cost while maintaining high security. Therefore, the use of semi-quantum cryptographic protocols in smart grids can not only improve the security of the system but also reduce implementation costs and technical barriers, thereby promoting widespread application in practical scenarios. As shown in Figure 1, the Control Center (CC) is situated at the core of the smart grid’s fundamental structure. It functions as the central hub that oversees, manages, and makes decisions within the power system. The Neighborhood Gateway (NG) serves a crucial role as middleware, facilitating the transmission of information from the power system and connecting terminal equipment with upper-layer systems. Security concerns between the CC and NG directly affect the overall stability of the power system [29,30,31,32]. Therefore, this paper proposes a semi-quantum two-way authentication technology that uses single photons for communication between the CC and the next NG. The CC has full quantum capabilities, while the NG has partial quantum capabilities, specifically using Z-based measurement particles, preparing Z-based particles, rearranging particle positions, and directly returning particles. The protocol is resistant to common attack methods such as double controlled NOT (CNOT) attacks, impersonation attacks, interception measurement retransmission attacks, and entanglement measurement attacks. Additionally, it significantly reduces the consumption of quantum resources and equipment requirements, effectively addressing the problem of identity forgery.

The rest of this paper is organized as follows: Section 2 describes the bidirectional authentication protocol flow in detail; Section 3 presents the security analysis of the protocol; Section 4 compares this protocol with other protocols; Section 5 provides an overview of the circuit simulation performed on the IBM platform; finally, Section 6 offers the conclusions.

## 2. Two-Way Authentication and Communication between NG and CC

**Figure 2:**
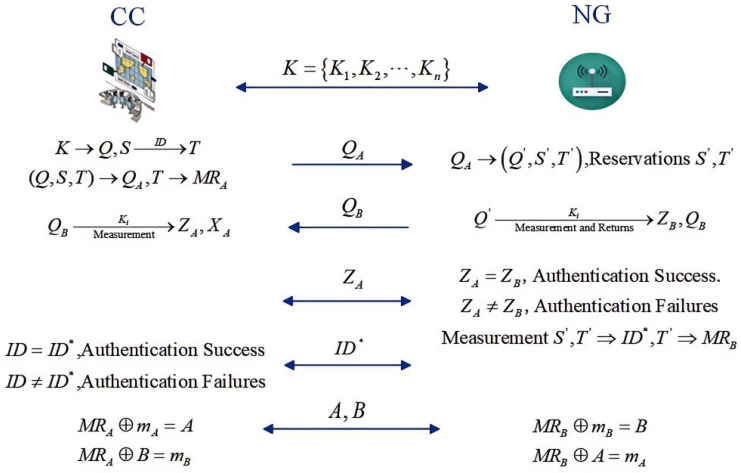
Protocol flow chart.

This section details the semi-quantum two-way authentication protocol between the NG and CC. The protocol comprises three discrete phases: initialization, authentication, and data transport. The specific process is shown in Figure 2.

### 2.1. Initialization Phase

Step 1: NG and CC share the key sequence $K=\{K_{1},K_{2},\cdots ,K_{n}\}$ through the semi-quantum key distribution protocol. Here, $K_{i}\in \left\{00,01,10,11\right\}$ for i=1,2,…,n.

Step 2: The CC generates *n* photon sequences $Q=\{Q_{1},Q_{2},\cdots ,Q_{n}\}$ according to Table 1, where $Q_{i}\in \left\{|0\rangle ,|1\rangle ,|+\rangle ,|-\rangle \right\}$ for i=1,2,…,n . Simultaneously, it randomly generates *n* photon sequences $S=\{S_{1},S_{2},\cdots ,S_{n}\}$ , where $S_{i}\in \left\{|0\rangle ,|1\rangle \right\}$ for i=1,2,…,n .

Step 3: The CC operates on each bit in $S_{i}$ to generate $T=T_{1},T_{2},\cdots ,T_{n}$ , denoted as $T_{i}=U_{i}S_{i}$ , where $U_{i}\in I,X$ . When $ID_{i}=0$ ( $ID_{i}=1$ ), $U_{i}=I$ ( $U_{i}=X$ ). Apply the encoding rule to generate the classic bit sequence $MR_{A}$ from *T*, where the encoding rule is: $|0\rangle$ represents 0, and $|1\rangle$ represents 1. CC rearranges the positions of *Q*, *S*, and *T* according to the shared key sequence *K* to form $Q_{A}$ , and CC transmits $Q_{A}$ to NG. The rearrangement rules are as follows: When $K_{i}=00$ or $K_{i}=01$ , CC inserts $T_{i}$ after $Q_{i}$ and $S_{i}$ before $Q_{i}$ . When $K_{i}=10$ or $K_{i}=11$ , CC inserts $T_{i}$ before $Q_{i}$ and $S_{i}$ after $Q_{i}$ .

To make the above steps clearer, assume n=4 , the key sequence K={00,01,10,11} , and the quantum bit sequence *Q*: $K_{1}=00\to Q_{1}=|0\rangle$ , $K_{2}=01\to Q_{2}=|1\rangle$ , $K_{3}=10\to Q_{3}=|+\rangle$ , $K_{4}=11\to Q_{4}=|-\rangle$ . Thus, $Q=\{|0\rangle ,|1\rangle ,|+\rangle ,|-\rangle \}$ . Assume $S=\{|0\rangle ,|1\rangle ,|0\rangle ,|1\rangle \}$ , ID={0,1,0,1} and generate the *T*: For $ID_{1}=0$ , $T_{1}=IS_{1}=|0\rangle$ . For $ID_{2}=1$ , $T_{2}=XS_{2}=X|1\rangle =|0\rangle$ . For $ID_{3}=0$ , $T_{3}=IS_{3}=|0\rangle$ . For $ID_{4}=1$ , $T_{4}=XS_{4}=X|1\rangle =|0\rangle$ . Therefore, $T=\{|0\rangle ,|0\rangle ,|0\rangle ,|0\rangle \}$ . Rearranging the positions: $K_{1}=00\to |S_{1},Q_{1},T_{1}\rangle =|0,0,0\rangle$ , $K_{2}=01\to |S_{2},Q_{2},T_{2}\rangle =|1,1,0\rangle$ , $K_{3}=10\to |T_{3},Q_{3},S_{3}\rangle =|0,+,0\rangle$ , $K_{4}=11\to |T_{4},Q_{4},S_{4}\rangle =|0,-,1\rangle$ . The final sequence $Q_{A}=\left\{|0,0,0\rangle ,|1,1,0\rangle ,|0,+,0\rangle ,|0,-,1\rangle \right\}$ is transmitted to NG.

### 2.2. Authentication Phase

Step 4: After receiving $Q_{A}$ , NG uses the shared key sequence *K* and the aforementioned arrangement rules to reconstruct $Q^{\prime }$ , $S^{\prime }$ , and $T^{\prime }$ . NG retains $S^{\prime }$ and $T^{\prime }$ , and then proceeds with the following operations on $Q^{\prime }$ :

When $K_{i}=00/01$ , NG measures ${Q_{i}}^{\prime }$ based on *Z*, records the measurement result as $Z_{B}$ , generates the same state as the measurement result, and returns it to CC.

When $K_{i}=10/11$ , NG immediately sends ${Q_{i}}^{\prime }$ back to CC without performing any additional processing. All photons that are returned by NG are labeled $Q_{B}$ .

Step 5: After receiving $Q_{B}$ , CC performs the following operations based on the key *K*:

If $K_{i}=00/01$ , CC calculates ${Q_{B}}_{i}$ based on *Z* and records the result as $Z_{A}$ .

If $K_{i}=10/11$ , CC calculates ${Q_{B}}_{i}$ based on *X* and records the result as $X_{A}$ . Subsequently, CC verifies whether $Z_{A}$ and $X_{A}$ are derived from the key *K* to ensure the security of the channel, and then announces the value of $Z_{A}$ . Specifically, according to Table 1, if $K_{i}=10$ , then ${X_{A}}_{i}=|+\rangle$ ; if $K_{i}=11$ , then ${X_{A}}_{i}=|-\rangle$ ; similarly, if $K_{i}=00$ , then ${Z_{A}}_{i}=|0\rangle$ ; if $K_{i}=01$ , then ${Z_{A}}_{i}=|1\rangle$ . If the above conditions are met, the authentication channel is considered secure, and CC then announces $Z_{A}$ . Otherwise, it is considered that there is an eavesdropper in the channel, and the protocol is terminated and restarted.

Step 6: NG compares the value of $Z_{B}$ with the value announced by CC. The authentication procedure will fail if $Z_{A}\neq Z_{B}$ . If $Z_{A}=Z_{B}$ , NG will successfully verify CC. Subsequently, NG measures the values of $T^{\prime }$ and $S^{\prime }$ based on *Z*, documents the results as $Z_{T}$ and $Z_{S}$ , compares them to determine $ID^{*}$ , and ultimately announces $ID^{*}$ .

Step 7: CC compares the value of ID with the value announced by NG. If $ID\neq ID^{*}$ , the authentication process will fail. If $ID=ID^{*}$ , CC will successfully verify NG.

### 2.3. Data Transport Phase

Step 8: After mutual authentication, NG stores the measurement result $Z_{T}$ as the classic bit sequence $MR_{B}$ , where $|0\rangle$ represents 0, and $|1\rangle$ represents 1. NG then performs an XOR operation on its own data information $m_{B}$ and $MR_{B}$ , obtaining $B=m_{B}⊕MR_{B}$ .

Step 9: Similarly, CC performs an XOR operation on its data information $m_{A}$ and $MR_{A}$ to produce $A=m_{A}⊕MR_{A}$ . CC and NG then declare *A* and *B*. Throughout this procedure, CC can determine the information of NG’s data $m_{B}$ by calculating $m_{B}=B⊕MR_{A}$ . Similarly, NG can determine the data information $m_{A}$ of CC by calculating $m_{A}=A⊕MR_{B}$ .

## 3. Security Analysis

The primary security concern during the identity authentication process is the possibility of identity forgery. To successfully form an identity, the attacker must have knowledge of the shared key sequence *K* used by the participants. The potential attacker in this case is an external threat known as Eve. Eve can employ various attack strategies, including double CNOT attacks, impersonation attacks, intercept-measure-resend attacks, entanglement measurement attacks, etc. to obtain the key sequence *K*. In this section, we perform a thorough analysis to determine whether Eve can illegitimately fabricate an identity by acquiring the shared key sequence *K* through the mentioned attack strategies, bypassing the identity authentication process successfully.

### 3.1. Double CNOT Attack

Eve can execute a double CNOT attack to gather information about the photons in transit, thereby acquiring the shared key sequence *K*. To carry out this assault, Eve prepares the auxiliary quantum state ${{|q\rangle }_{e}}_{i}$ to perform the CNOT operation on each photon in ${Q_{A}}_{i}$ and ${Q_{B}}_{i}$ . Here, the ${{|q\rangle }_{e}}_{i}$ is used as the target qubit, while ${Q_{A}}_{i}$ and ${Q_{B}}_{i}$ are used as the control qubits.

Eve intercepts the quantum state $Q_{A_{i}}$ that is transmitted from the CC to the NG in Step 3. Afterward, the CNOT operation is performed on each photon in the quantum register $Q_{A}$ , denoted as $U_{CNOT}{Q_{A}}_{i}$ . To be more precise:

(1) $$\begin{array}{cc} \; & U_{CNOT}{|0\rangle }_{A}⊗{|q\rangle }_{e}={|0\rangle }_{A}⊗{|q\rangle }_{e}. \end{array}$$

(2) $$\begin{array}{cc} \; & U_{CNOT}{|1\rangle }_{A}⊗{|q\rangle }_{e}={|1\rangle }_{A}⊗{|\bar{q}\rangle }_{e}. \end{array}$$

(3) $$\begin{array}{cc} \; & U_{CNOT}{|+\rangle }_{A}⊗{|q\rangle }_{e}=\frac{1}{\sqrt{2}}\left({|0q\rangle }_{Ae}+{|1\bar{q}\rangle }_{Ae}\right). \end{array}$$

(4) $$\begin{array}{cc} \; & U_{CNOT}{|-\rangle }_{A}⊗{|q\rangle }_{e}=\frac{1}{\sqrt{2}}\left({|0q\rangle }_{Ae}-{|1\bar{q}\rangle }_{Ae}\right). \end{array}$$

After performing the CNOT operation, the state of the qubit $Q_{A}$ is updated and recorded as ${Q_{A}}^{\prime }$ . Eve transmits ${Q_{A}}^{\prime }$ to NG. NG receives ${Q_{A}}^{\prime }$ and performs in Step 4 to generate ${Q_{B}}^{\prime }$ based on the shared key *K* and sends it to CC. Eve intercepts ${Q_{B}}^{\prime }$ and performs a CONT operation on each photon in ${Q_{B}}^{\prime }$ , following these steps:

(5) $$\begin{array}{cc} \; & U_{CNOT}\left({|0\rangle }_{B}⊗{|q\rangle }_{e}\right)={|0\rangle }_{B}⊗{|q\rangle }_{e}. \end{array}$$

(6) $$\begin{array}{cc} \; & U_{CNOT}\left({|1\rangle }_{B}⊗{|\bar{q}\rangle }_{e}\right)={|1\rangle }_{B}⊗{|q\rangle }_{e}. \end{array}$$

(7) $$\begin{array}{cc} \; & U_{CNOT}\left(\frac{1}{\sqrt{2}}\left({|0q\rangle }_{Be}+{|1\bar{q}\rangle }_{Be}\right)\right)={|+\rangle }_{B}⊗{|q\rangle }_{e}. \end{array}$$

(8) $$\begin{array}{cc} \; & U_{CNOT}\left(\frac{1}{\sqrt{2}}\left({|0q\rangle }_{Be}-{|1\bar{q}\rangle }_{Be}\right)\right)={|-\rangle }_{B}⊗{|q\rangle }_{e}. \end{array}$$

The above formula shows that the quantum state ${|q\rangle }_{e}$ remains unchanged. Eve is unable to carry out a measurement on the auxiliary qubit to gather information about the specific transporting photon. Thus, when Eve executes the attack, it is only feasible to discern the exact state of the moving photon by probabilistic means, and no valuable information can be acquired.

### 3.2. Impersonation Attack

**Figure 3:**
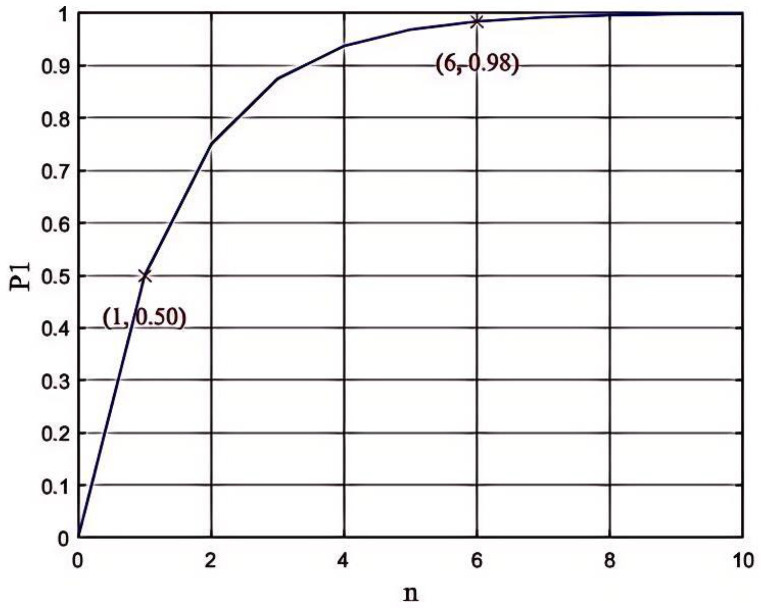
Detection probability of Eve impersonating CC.

If the attacker Eve impersonates NG or CC, she will try to complete fake authentication by randomly preparing qubits, sending qubit sequence, and performing single-qubit measurement. Supposing that Eve attempts to mimic CC, she stochastically chooses and creates 3n qubits from the set $\left\{|0\rangle ,|1\rangle ,|+\rangle ,|-\rangle \right\}$ to construct $Q_{A}$ , which she subsequently transmits to NG. NG performs the same action as explained in Step 4 and sends $Q_{B}$ to Eve. Due to Eve’s lack of knowledge of the shared key sequence *K*, she is unable to accurately determine the value of $Z_{A}$ . If Eve successfully authenticates, the condition $Z_{A}=Z_{B}$ must be met, where $Z_{A}\in {\left\{0,1\right\}}^{n}$ . Consequently, the probability of Eve’s successful authentication is ${\left(\frac{1}{2}\right)}^{n}$ , and the probability of authentication failure is denoted as $P_{1}=1-{\left(\frac{1}{2}\right)}^{n}$ . From Figure 3, if *n* is large enough, $P_{1}$ is approximate to 1. Consequently, NG’s attempt to authenticate Eve will fail.

**Figure 4:**
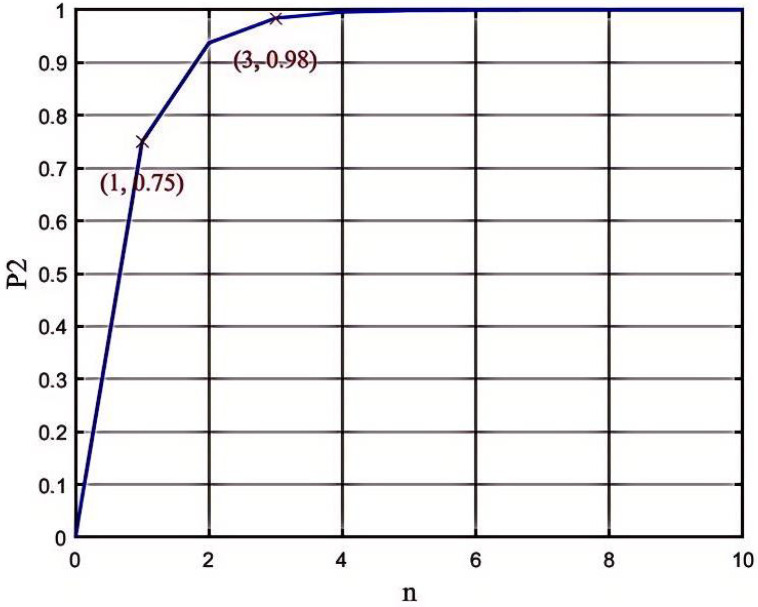
Detection probability of Eve impersonating NG.

Supposing that Eve attempts to mimic NG, Eve’s lack of knowledge about the key sequence *K* prevents her from recovering $Q_{1}$ , $S_{1}$ , and $T_{1}$ based on *K*. Instead, she can only generate *n* photon sequences $Q_{B}$ by randomly selecting from the set $\left\{|0\rangle ,|1\rangle ,|+\rangle ,|-\rangle \right\}$ and sending them to CC. To successfully obtain CC certification, Eve must have a value of $Q_{B}$ that is precisely identical to the value of *Q*. Eve must have precise knowledge of the exact state of every photon in *Q*. The possible states of each photon in *Q* are limited to $\left\{|0\rangle ,|1\rangle ,|+\rangle ,|-\rangle \right\}$ . Therefore, the probability of Eve passing the authentication is ${\left(\frac{1}{4}\right)}^{n}$ , the probability that Eve will fail to imitate CC authentication is $P_{2}=1-{\left(\frac{1}{4}\right)}^{n}$ , as the number of photons *n* increases, the detection probability $P_{2}$ tends towards 1. Figure 4 illustrates the correlation between the quantity of photons *n* needed to counteract this assault and the probability.

### 3.3. Intercept-Measure-Resend Attack

To acquire the shared key sequence *K* between the NG and the CC, Eve employs an intercept-measure-resend attack. In Step 3, Eve intercepts and measures the value of $Q_{A}$ . Subsequently, she constructs a fresh sequence of photons, denoted as ${Q_{A}}^{\prime }$ , utilizing the acquired measurement results. Finally, Eve transmits this new sequence to Subsequently, at Step 4, Eve intercepts $Q_{B}$ , performs similar measurements to generate $Q_{B}^{\prime }$ , and sends it to CC. Without knowing the shared key sequence *K* between the CC and the NG, Eve cannot determine the original location of $Q_{B}$ . Therefore, Eve cannot gain any valuable knowledge. After receiving ${Q_{B}}^{\prime }$ , CC utilizes the shared key sequence *K* to measure ${Q_{B}}^{\prime }$ and authenticate its security. The possible states of each photon in ${Q_{B}}^{\prime }$ are limited to $\left\{|0\rangle ,|1\rangle ,|+\rangle ,|-\rangle \right\}$ . The probability of Eve successfully passing the inspection is ${\left(\frac{1}{4}\right)}^{n}$ , while the probability of the CC detecting Eve’s attack is $P_{3}=1-{\left(\frac{1}{4}\right)}^{n}$ . As the number of photons *n* increases, the detection probability $P_{3}$ tends toward 1. It can be inferred that Eve’s presence can be discovered when executing an intercept-measure-resend attack.

### 3.4. Entanglement Measurement Attack

We assume that Eve uses the auxiliary state $|e\rangle$ to carry out the entanglement attack. Eve captures the photon sent from CC to NG, applies *U* operation to the captured photon and auxiliary state $|e\rangle$ , and then sends the modified photon to NG. After NG returns the photon to CC, Eve measures her auxiliary state $|e\rangle$ to obtain information about the key sequence *K*, as follows:

Suppose that Eve employs the auxiliary state $|e\rangle$ to carry out the entanglement assault. Eve captures the photons sent from CC to NG, applies *U* operations to both the captured photons and the auxiliary state $|e\rangle$ , and then sends the modified photons to NG. After NG returns the photon to CC, Eve measures her auxiliary state $|e\rangle$ in order to obtain relevant information about the key sequence *K*, as described below:

(9) $$\begin{array}{cc} \; & U|0\rangle |e\rangle =\alpha |0\rangle |e_{00}\rangle +\beta |1\rangle |e_{01}\rangle . \end{array}$$

(10) $$\begin{array}{cc} \; & U|1\rangle |e\rangle =\varepsilon |0\rangle |e_{10}\rangle +\delta |1\rangle |e_{11}\rangle . \end{array}$$

where ${\left|\alpha \right|}^{2}+{\left|\beta \right|}^{2}=1$ and ${\left|\varepsilon \right|}^{2}+{\left|\delta \right|}^{2}=1$ .

(11) $$\begin{array}{c} \begin{array}{c} U|+\rangle |e\rangle =\frac{1}{2}\left(|+\rangle \left(\alpha |e_{00}\rangle +\beta |e_{01}\rangle \right)\right)\\ +\frac{1}{2}\left(|+\rangle \left(\varepsilon |e_{10}\rangle +\delta |e_{11}\rangle \right)\right)\\ +\frac{1}{2}\left(|-\rangle \left(\alpha |e_{00}\rangle -\beta |e_{01}\rangle \right)\right)\\ +\frac{1}{2}\left(|-\rangle \left(\varepsilon |e_{10}\rangle -\delta |e_{11}\rangle \right)\right).\; \end{array} \end{array}$$

(12) $$\begin{array}{c} \begin{array}{c} U|-\rangle |e\rangle =\frac{1}{2}\left(|+\rangle \left(\alpha |e_{00}\rangle +\beta |e_{01}\rangle \right)\right)\\ -\frac{1}{2}\left(|+\rangle \left(\varepsilon |e_{10}\rangle +\delta |e_{11}\rangle \right)\right)\\ +\frac{1}{2}\left(|-\rangle \left(\alpha |e_{00}\rangle -\beta |e_{01}\rangle \right)\right)\\ +\frac{1}{2}\left(|-\rangle \left(\varepsilon |e_{10}\rangle -\delta |e_{11}\rangle \right)\right). \end{array} \end{array}$$

where ${\left|\alpha \right|}^{2}+{\left|\beta \right|}^{2}=1$ and ${\left|\varepsilon \right|}^{2}+{\left|\delta \right|}^{2}=1$ . The photon statement transmitted from the CC to the NG can be found in one of the following states: |0〉 , |1〉 , |+〉 or |−〉 . To avoid detection, Eve had to ensure that both β and ε were equal to 0.

(13) $$\begin{array}{cc} \; & \alpha |e_{00}\rangle -\beta |e_{01}\rangle +\varepsilon |e_{10}\rangle -\delta |e_{11}\rangle =0. \end{array}$$

(14) $$\begin{array}{cc} \; & \alpha |e_{00}\rangle +\beta |e_{01}\rangle -\varepsilon |e_{10}\rangle -\delta |e_{11}\rangle =0. \end{array}$$

At this time, $\alpha |e_{00}\rangle =\delta |e_{11}\rangle$ . This means that Eve cannot distinguish between $\alpha |e_{00}\rangle$ and $\delta |e_{11}\rangle$ . Therefore, entanglement measurement attacks can be resisted.

## 4. Efficiency Analysis

In this section, we define the quantum bit efficiency as [33] $\eta =\frac{b_{s}}{q_{t}+b_{t}}$ , where $b_{s}$ represents the expected bits obtained after consuming quantum bits in the protocol, $q_{t}$ represents the quantum bits consumed in the protocol, and $b_{t}$ represents the bits consumed using the classical channel. In this protocol, CC generates 3n -bit single particles in the initial stage, NG measures and generates n/2 -bit single particles in the authentication stage, while the CC and NG share 2n -bit key sequence. The values $Z_{A}$ and $Z_{B}$ used for authentication are n/2 -bit, $ID^{*}$ is *n*-bit, $MR_{A}$ and $MR_{B}$ used for data transmission are *n*-bit. Therefore, the quantum bit efficiency of the protocol is $\eta =\frac{n/2+n+n}{3n+n/2+2n}\times 100\%\approx 45.5\%$ .

This section compares the proposed protocol with previous protocols in terms of quantum resources, involvement of third parties, bits of shared keys, bidirectional authentication, and quantum bit efficiency, as illustrated in Table 2.

According to Table 2, this protocol has several advantages over previous ones. First, it uses single particles as quantum resources, which are simpler to implement compared to protocols that require more complex entangled states, such as Bell states or GHZ states. Second, it does not require the participation of a third party, which not only enhances security but also reduces potential points of failure. Additionally, the protocol achieves a quantum bit efficiency of 45.5%, which is significantly higher than that of other protocols. Finally, the protocol supports two-way authentication, providing a more secure communication channel. These advantages make the protocol more attractive and practical in real-world applications.

## 5. Simulation Experiments on IBM Platform

Simulating circuits serves multiple purposes, such as elucidating protocol fundamentals, validating correctness, and affirming feasibility through tangible examples of communication processes. Based on the protocol described in Section 2, we can follow the steps below to simulate its various stages on the IBM Quantum Cloud Platform, explaining the role and results of each step.

**Figure 5:**
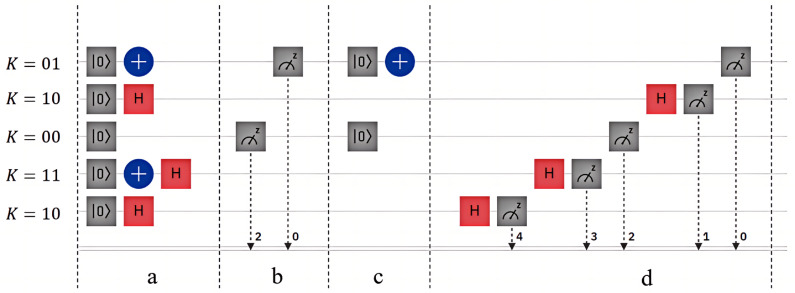
Quantum circuit diagram for CC authenticate NG. The diagram in (**a**) shows the quantum state generation by CC, while diagrams (**b**,**c**) depict the process where NG measures the quantum state based on *K* and generates the same quantum state according to the measurement results. Diagram (**d**) illustrates the circuit where CC measures the quantum state sequence returned by NG.

**Figure 6:**
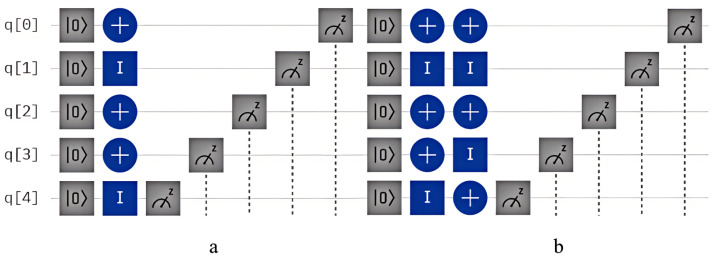
Quantum circuit diagram for NG authenticate CC.Diagram (**a**) shows the circuit for generating the quantum state sequence *S* by CC, while diagram (**b**) illustrates the circuit for generating the quantum state sequence *T* by NG.

Assuming the shared key sequence K:{10,11,00,10,01} , according to Table 1, the Control Center (CC) generates the quantum state sequence $Q:\{|+\rangle ,|-\rangle ,|0\rangle ,|+\rangle ,|1\rangle \}$ , the specific quantum circuit is depicted in Figure 5a. Additionally, CC randomly generates the sequences $S:\{|0\rangle ,|1\rangle ,|1\rangle ,|0\rangle ,|1\rangle \}$ and ID:{10101} , the specific quantum circuit for this is shown in Figure 6a. Based on the ID sequence, it generates $T:\{|1\rangle ,|1\rangle ,|0\rangle ,|0\rangle ,|0\rangle \}$ , the specific quantum circuit for *T* is shown in Figure 6b. Then CC rearranges *Q*, *S* and *T* based on the shared key *K* to generate $Q_{A}$ : $\left\{|+\rangle ,|0\rangle ,|1\rangle ,|-\rangle ,|1\rangle ,|1\rangle ,|1\rangle ,|0\rangle ,|+\rangle ,|0\rangle ,|0\rangle ,|1\rangle ,|0\rangle \right\}$ and transmits it to NG.

**Figure 7:**
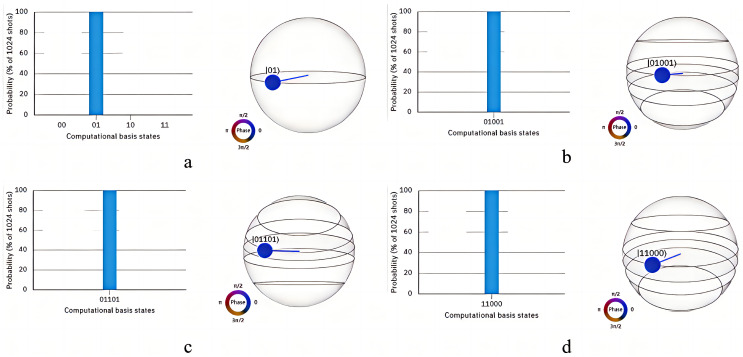
Measurement results. In (**a**), the diagram shows the measurement results of NG as illustrated in Figure 5b. Diagram (**b**) represents the measurement results of CC as shown in Figure 5d. Diagrams (**c**,**d**) depict the measurement results of NG as illustrated in Figure 6a,b.

After receiving $Q_{A}$ , NG reconstructs $Q^{\prime }$ , $S^{\prime }$ , and $T^{\prime }$ according to the key sequence *K*. It measures $Q^{\prime }$ and generates a quantum state identical to the measurement result, the specific quantum circuit is depicted in Figure 5b,c and the measurement result is illustrated in Figure 7a. Subsequently, CC measures the particle returned by NG according to the same key sequence *K*, the specific quantum circuit is shown in Figure 5d, and measurement result is displayed in Figure 7b.

From the quantum circuit diagrams and measurement results, it is evident that in NG’s measurements, the position of C[2] in the classical memory is 0, and C[0] ’s position is 1, denoted as $Z_{B}:\left\{0,1\right\}$ . In contrast, in CC’s measurements, the positions of C[2] and C[0] are 0 and 1 respectively, denoted as $Z_{A}:\left\{0,1\right\}$ . Thus, $Z_{A}=Z_{B}$ , indicating NG successfully authenticates CC. NG then measures $S^{\prime }$ and $T^{\prime }$ , the specific quantum circuit is shown in Figure 6. From Figure 7c,d, it is observed that $Z_{S}$ : {01101} and $Z_{T}$ : {11000} , confirming $ID^{*}:\left\{10101\right\}$ . At this point, $ID=ID^{*}$ , indicating CC successfully authenticates NG.

The security and reliability of smart grids represent significant challenges in modern power systems. Through the simulation process outlined above, it is evident that the proposed protocol utilizes fundamental principles of quantum mechanics to ensure secure key distribution. By integrating quantum states with classical information, the protocol authenticates the identities of communicating parties, verifies data integrity and authenticity, prevents transmission tampering, and effectively mitigates man-in-the-middle attacks. Moreover, the protocol’s steps for reconstruction and measurement effectively counteract errors induced by environmental noise, thereby enhancing system reliability. In terms of practicality, the advancement of quantum computing technology provides a solid technical foundation for implementing this authentication protocol. As a semi-quantum authentication method, it notably reduces equipment requirements and resource consumption. In conclusion, this protocol not only addresses practical challenges in smart grid security but also delivers dependable security assurances for future developments in power systems.

## 6. Conclusions

In this paper, a new two-way authentication protocol is proposed to protect the power grid industry against potential threats from quantum computers. Compared with traditional methods, the proposed semi-quantum protocol leverages quantum principles while minimizing the need for quantum resources. This approach offers a practical solution for smart grids, enhancing security without requiring major modifications to existing infrastructure. A comparison with existing protocols shows that the proposed protocol has high quantum bit efficiency, making it more attractive and feasible for practical applications. Security analysis demonstrates that the protocol can resist common attack strategies and ensure the integrity of communications. Circuit simulations were performed on an IBM platform to verify the theoretical framework, confirm the feasibility of the protocol, and ensure its compliance with quantum principles. It is important to emphasize that the proposed protocol can be implemented with existing technologies.

## Figures and Tables

**Table 1:** Rules for generating quantum bit sequences $Q_{i}$ from shared key sequences $K_{i}$ .

$K_{i}$	$Q_{i}$
00	$|0\rangle$
01	$|1\rangle$
10	$|+\rangle$
11	$|-\rangle$

**Table 2:** Protocol comparison table.

	Ref. [24]	Ref. [25]	Ref. [26]	Ref. [27]	Ref. [28]	Our
Quantum resources	Single Particle	Bell state	GHZ state	Bell state	Single Particl	Single Particle
Third-party participation	No	Yes	No	Yes	No	No
Shared key bits	2n	2n	5n	2n	2n	2n
Two-way authentication	No	Yes	No	Yes	No	Yes
Quantum bit efficiency	20%	25%	10%	14.2%	12.5%	45.5%

## Data Availability

This manuscript has no associated data. No public involvement in any aspect of this research, AI or AI-assisted tools were not used in drafting any aspect of this manuscript.

## Abbreviations

The following abbreviations are used in this manuscript:

NG	Neighborhood Gateway
CC	Control Center
QKD	Quantum Key Distribution
IBM	International Business Machines
TLS	Transport Layer Security
SSL	Secure Sockets Layer

## References

[1] Alotaibi I., Abido M.A., Khalid M., Savkin A.V. (2020). A comprehensive review of recent advances in smart grids: A sustainable future with renewable energy resources. Energies.

[2] Islam M.A., Hasanuzzaman M., Rahim N.A., Nahar A., Hosenuzzaman M. (2014). Global Renewable Energy-Based Electricity Generation and Smart Grid System for Energy Security. Sci. World J..

[3] Yu X., Xue Y. (2016). Smart grids: A cyber–physical systems perspective. Proc. IEEE..

[4] Kimani K., Oduol V., Langat K. (2019). Cyber security challenges for IoT-based smart grid networks. Int. J. Crit. Infrastruct. Prot..

[5] Otuoze A.O., Mustafa M.W., Larik R.M. (2018). Smart grids security challenges: Classification by sources of threats. J. Electr. Syst. Inf. Technol..

[6] Obaidat M.A., Obeidat S., Holst J., Al Hayajneh A. (2020). A comprehensive and systematic survey on the internet of things: Security and privacy challenges, security frameworks, enabling technologies, threats, vulnerabilities and countermeasures. Computers.

[7] Islam S.N., Baig Z., Zeadally S. (2019). Physical layer security for the smart grid: Vulnerabilities, threats, and countermeasures. IEEE Trans. Ind. Inform..

[8] Cavaliere F., Mattsson J., Smeets B. (2020). The security implications of quantum cryptography and quantum computing. Netw. Secur..

[9] Mitra S., Jana B., Bhattacharya S., Pal P., Poray J. (2017). Quantum cryptography: Overview, security issues and future challenges. Proceedings of the 2017 4th International Conference on Opto-Electronics and Applied Optics (Optronix).

[10] Zhou N.R., Zhang T.F., Xie X.W. (2023). Hybrid quantum–classical generative adversarial networks for image generation via learning discrete distribution. Signal Process. Image Commun..

[11] Alshowkan M., Evans P.G., Starke M., Earl D., Peters N.A. (2022). Authentication of smart grid communications using quantum key distribution. Sci. Rep..

[12] Li Y., Zhang P., Huang R. (2019). Lightweight quantum encryption for secure transmission of power data in smart grid. IEEE Access.

[13] Singhrova A. (2023). Quantum Key Distribution-based Techniques in IoT. Sci. Temper.

[14] Wang W., Lu Z. (2013). Cyber security in the smart grid: Survey and challenges. Comput. Netw..

[15] Fouda M.M., Fadlullah Z.M., Kato N., Lu R., Shen X.S. (2011). A lightweight message authentication scheme for smart grid communications. IEEE Trans. Smart Grid.

[16] Crépeau C., Salvail L. (1995). Quantum oblivious mutual identification. Entropy.

[17] Song Y., Wu Y., Wu S., Li D., Wen Q., Qin S., Gao F. (2024). A quantum federated learning framework for classical clients. Sci. China-Phys. Mech. Astron..

[18] Zawadzki P. (2019). Quantum identity authentication without entanglement. Quantum Inf. Process..

[19] Termos H. (2024). Quantum Authentication Evolution: Novel Approaches for Securing Quantum Key Distribution. Entropy..

[20] Shi W.M., Zhang J.B., Zhou Y.H., Yang Y.G. (2015). A novel quantum deniable authentication protocol without entanglement. Quantum Inf. Process..

[21] Boyer M., Kenigsberg D., Mor T. Quantum key distribution with classical Bob. Proceedings of the 2007 First International Conference on Quantum, Nano, and Micro Technologies (ICQNM’07).

[22] Krawec W.O. Security proof of a semi-quantum key distribution protocol. Proceedings of the 2015 IEEE International Symposium on Information Theory (ISIT).

[23] Iqbal H., Krawec W.O. (2020). Semi-quantum cryptography. Quantum Inf. Process..

[24] Zhou N.R., Zhu K.N., Bi W., Gong L.H. (2019). Semi-quantum identification. Quantum Inf. Process..

[25] Zhang S., Chen Z.K., Shi R.H., Liang F.Y. (2020). A novel quantum identity authentication based on Bell states. Int. J. Theor. Phys..

[26] Wang H.W., Tsai C.W., Lin J., Yang C.W. (2022). Authenticated semi-quantum key distribution protocol based on W states. Sensors.

[27] Dutta A., Pathak A. (2022). Controlled secure direct quantum communication inspired scheme for quantum identity authentication. Quantum Inf. Process..

[28] Yang C.W., Wang H.W., Lin J., Tsai C.W. (2023). Semi-Quantum Identification without Information Leakage. Mathematics.

[29] Iqbal H., Krawec W.O. (2020). High-dimensional semiquantum cryptography. IEEE Trans. Quantum Eng..

[30] Wu J., Ma Q., Deng X., Qin Z. Lightweight authentication for smart metering infrastructure in smart grid. Proceedings of the International Conference on Cyber Security, Artificial Intelligence, and Digital Economy (CSAIDE 2023).

[31] Ferrag M.A., Maglaras L.A., Janicke H., Jiang J., Shu L. (2018). A systematic review of data protection and privacy preservation schemes for smart grid communications. Sustain. Cities Soc..

[32] Saxena N., Choi B.J. (2015). State of the art authentication, access control, and secure integration in smart grid. Energies.

[33] Cabello A. (2000). Quantum key distribution in the Holevo limit. Phys. Rev. Lett..

