# Correction: Zhang et al. A New Semi-Quantum Two-Way Authentication Protocol between Control Centers and Neighborhood Gateways in Smart Grids. *Entropy* 2024, *26*, 644

**DOI:** 10.3390/e26100873

**Published:** 2024-10-18

**Authors:** Qiandong Zhang, Kejia Zhang, Kunchi Hou, Long Zhang

**Affiliations:** 1 School of Mathematical Science, Heilongjiang University, Harbin 150080, China; qiandongzhang@163.com (Q.Z.); houkunchi@hlju.edu.cn (K.H.); lzhang@hlju.edu.cn (L.Z.); 2 State Key Laboratory of Public Big Data, Guizhou University, Guiyang 550000, China

In the published publication [1], there was an error regarding the email address for Qiandong Zhang. The correct email address should be qiandongzhang@163.com.

There was an error in the original publication [1]. Corrections have been made to recognize the work of Mitra et al. [2] and Song et al. [3] in the References Section in Ref. 9 and Ref. 17, respectively.

The authors state that the scientific conclusions are unaffected. This correction was approved by the Academic Editor. The original publication has also been updated.
